# Symptom heterogeneity in students with mild to severe depression symptomatology and their differential symptom-specific changes during an internet-based, guided cognitive behavioural therapy intervention

**DOI:** 10.1016/j.invent.2025.100834

**Published:** 2025-05-16

**Authors:** Lynn Boschloo, Jasmijn Wijnands, Nadia Garnefski, Vivian Kraaij, Petra Hurks, Danielle Remmerswaal, Reinout W. Wiers, Sascha Struijs, Elske Salemink

**Affiliations:** a Department of Clinical Psychology, Utrecht University, Utrecht, the Netherlands; b Division of Clinical Psychology, Leiden University, Leiden, the Netherlands; c Faculty of Psychology and Neuroscience, Maastricht University, Maastricht, the Netherlands; d Department of Psychology, Education, and Child Studies, Erasmus University, Rotterdam, the Netherlands; e Addiction Development and Psychopathology (ADAPT) lab, Department of Psychology, Centre for Urban Mental Health, University of Amsterdam, Amsterdam, the Netherlands; f Department of Clinical Psychology, VU University, Amsterdam, the Netherlands

**Keywords:** University students, Depression symptoms, Perceived stress, Quality of life, Internet-based CBT, Network estimation techniques

## Abstract

**Background:**

Students often report depression and stress symptomatology but may differ in their symptoms and their symptom-specific changes during interventions. This study adopted a symptom-specific approach and examined 1) individual symptoms in students experiencing mild to severe depression symptomatology and 2) changes in individual symptoms during a guided, internet-based intervention. We zoomed in on how these (changes in) symptoms were related to each other and to (changes in) overall quality of life.

**Methods:**

This study included 1816 students with mild to severe baseline depression symptomatology, of which 412 activated their account for an eight-week, guided, internet-based Cognitive Behavioural Therapy intervention (*Moodpep*) and completed the post-treatment assessment. Depression symptomatology was assessed with the Patient Health Questionnaire, stress symptomatology with the Perceived Stress Scale and overall quality of life with a single item from the Mental Health Quality of Life questionnaire. Network estimations were conducted to examine the interrelations of (changes in) symptoms.

**Results:**

Mean scores of baseline symptoms differed substantially, and network estimations showed multiple positive connections across symptoms and negative connections of symptoms with overall quality of life. During the intervention, all symptoms reduced significantly, although with differential magnitude, and network estimations showed that changes in symptoms were differentially related to other changes in symptoms and changes in overall quality of life.

**Conclusions:**

Our findings highlight the importance of considering individual symptoms and their interrelations as a more complete and nuanced measure for 1) the heterogeneity of baseline symptomatology and 2) the heterogeneity of changes in symptomatology during an intervention.

## Highlights

• Students often report depression and related symptoms.
• Frequencies of these individual symptoms differ substantially.
• Moodpep is a guided iCBT intervention for students with depression symptomatology.
• Students using this intervention show medium reductions in symptomatology.
• Effect sizes differ considerably across individual symptoms.

## 1 Introduction

### 1.1 Depression symptomatology in students

In recent years, there has been a gradual increase in the prevalence of depression symptomatology among university students (Auerbach et al., 2018; Lipson et al., 2022; Storrie et al., 2010). The number of students with depression, alongside other mental health issues, has nearly doubled since 2013 (Lipson et al., 2022). According to a recent study including 10,000 university students, approximately one-third of students screened positive for one or more mental health disorders, with depression being the most common one (Auerbach et al., 2018). Depression symptomatology is also related to other adverse outcomes such as higher levels of perceived stress (Hewitt et al., 1992; Ramezankhani et al., 2013; Besirli et al., 2021) as well as impairments in quality of life (Gan and Yuen Ling, 2019; Jenkins et al., 2021). Consequently, research on prevention strategies has great potential in ultimately improving students' mental health.

### 1.2 Internet-based cognitive behavioural therapy for students with depression symptomatology

Internet-based cognitive behavioural therapy (iCBT), which focuses on changing negative cognitions and maladaptive behaviours, is a promising tool in providing early intervention to students with mental health problems (Day et al., 2013; Mitchell and Dunn, 2007; Mullin et al., 2015). Multiple randomized controlled trials have demonstrated the effectiveness of iCBT in reducing depression symptomatology among a broad range of populations (Andersson et al., 2014; Păsărelu et al., 2017; Van Luenen et al., 2018) including students (Day et al., 2013; Mitchell and Dunn, 2007; Mullin et al., 2015). Recent studies suggest that iCBT, especially with therapeutic guidance (i.e., guided iCBT), may be equally effective as face-to-face CBT while providing additional benefits such as convenience and privacy for participants (Andrews et al., 2018; Cuijpers et al., 2019b; Karyotaki et al., 2021; Kambeitz-Ilankovic et al., 2002). The *Moodpep* intervention is an iCBT program that was specifically designed for students with mild to moderate levels of depression and a pilot study (*N* = 31) showed large reductions in overall depression symptomatology in participating students (Garnefski and Kraaij, 2023). However, replication is needed, especially in studies with larger samples that not only consider depression symptomatology but also other outcomes.

### 1.3 The importance of a symptom-specific approach

In most trials evaluating depression interventions, including the study on the *Moodpep* intervention (Garnefski and Kraaij, 2023), therapeutic effects are typically evaluated using symptom sum scores to measure overall depression symptomatology (Borsboom and Cramer, 2013; Fried and Nesse, 2015). However, there is strong empirical reason to assume that depression is a heterogenous collection of symptoms (Fried and Nesse, 2015) and that these symptoms respond differently to a specific treatment (Bekhuis et al., 2018; Borsboom and Cramer, 2013; Cramer et al., 2016; Boschloo et al., 2019a, Boschloo et al., 2019b, Boschloo et al., 2022, Boschloo et al., 2023; Fried and Cramer, 2017). A recent study, for example, showed that depression symptoms vary substantially in adults with mild to moderate depression symptomatology and that, in addition, these symptoms exhibit varying responses to an iCBT intervention relative to care as usual (Boschloo et al., 2019a). Participants, for example, reported higher frequencies of symptoms such as ‘feeling tired or having little energy’ and ‘sleeping problems’ at baseline, whereas the lowest frequencies were found for ‘suicidal thoughts’ and ‘psychomotor agitation/retardation’. The largest treatment effects were observed for the specific symptoms ‘concentration problems’, ‘feeling guilty/worthless’ and ‘feeling tired or having low energy’, while the smallest effects were observed for ‘psychomotor agitation/retardation’ and ‘overeating or poor appetite’. These findings underscore that the traditional approach of using symptom sum scores discards valuable information regarding the symptom heterogeneity in adults with mild to moderate depression symptomatology and their differential symptom-specific responses to an iCBT intervention. Adopting a symptom-specific approach could also have great value in research involving students and interventions that are specifically developed for this vulnerable group.

### 1.4 The value of a symptom networks

The network approach to psychopathology focuses specifically on the complex patterns in which individual symptoms are related (Borsboom, 2017; Fried et al., 2017) and has shown great value in shedding light on the symptom heterogeneity of depression and other psychopathology (e.g., Boschloo et al., 2015; Bekhuis et al., 2016). In recent years, this approach has also shown to be valuable in understanding the complex, symptom-specific responses to various depression treatments (Bekhuis et al., 2018; Boschloo et al., 2019a, Boschloo et al., 2019b, Boschloo et al., 2022, Boschloo et al., 2023). We found, for example, that the iCBT intervention for adults with mild to moderate depression symptomatology (see paragraph 1.3) was directly related to larger reductions in only four symptoms (direct effects; i.e., ‘concentration problems’, ‘feeling guilty/worthless’, ‘feeling tired and having little energy’ and ‘sleeping problems’), which were related to larger reductions in the five other depression symptoms (indirect effects; e.g., ‘feeling depressed/hopeless’ and ‘suicidal thoughts’) as compared to the care as usual condition (Boschloo et al., 2019a). In addition, network studies on other depression treatments found that reductions in depression symptoms were also related to other mental health indicators such as comorbid symptomatology (e.g., Boschloo et al., 2022, Boschloo et al., 2023). However, no previous study has used network estimations to unravel the symptom heterogeneity in a student population while simultaneously considering the heterogeneity of symptom changes during an iCBT intervention developed for this population.

### 1.5 Study aims

The current study had two main aims. Firstly, we focused on a sample of 1816 students reporting at least mild depression symptomatology and zoomed in on their symptom heterogeneity. We aimed to determine the mean frequencies of nine specific depression and ten specific stress symptoms (aim 1.1) and employed network estimation techniques to unravel how these symptoms were interrelated (aim 1.2). In a last step, we aimed to examine how these symptoms were related to overall quality of life (aim 1.3), as this would be valuable in identifying symptoms that were the most burdensome for students. Secondly, we zoomed in on the heterogeneity of symptom changes during the guided iCBT intervention *Moodpep* (Garnefski and Kraaij, 2023) and, consequently, selected the 412 students who activated their account for the intervention and completed the post-treatment assessment. We aimed to determine how the depression symptoms and perceived stress symptoms changed during the intervention (aim 2.1) and how these changes in symptoms were interrelated (aim 2.2). Lastly, we aimed to examine how these changes in symptoms were related to changes in overall quality of life (aim 2.3), so we could identify those symptom changes that were the most meaningful for students.

## 2 Methods

### 2.1 Design & ethics

This study is part of the Caring Universities project, a collaborative initiative involving nine Dutch universities. The project aims to capture quantitative data on students' mental health and to offer students free online services to improve their mental well-being (Vrije Universiteit Amsterdam, 2020; https://www.caring-universities.com). Among these services is the guided iCBT intervention *Moodpep*, designed for students with mild to moderate depression symptomatology (Garnefski and Kraaij, 2023). The Caring Universities project is part of the World Mental Health Surveys International College Student (WMH-ICS) initiative (Cuijpers et al., 2019a, Cuijpers et al., 2019b). Information about the study's aims and procedure was provided via the Caring Universities platform and all students provided digital informed consent before they could participate in the study. The Caring Universities project as well as the current study was approved by the Scientific and Ethical Review Board of the participating universities.

For the first set of research aims (i.e., aims 1.1–1.3), we used a cross-sectional design and included all students who screened positive for the *Moodpep* intervention at the baseline (i.e., pre-treatment) assessment. For the second set of research aims (i.e., aims 2.1–2.3), we used a one-group pretest-posttest design, including all students who activated their account for the *Moodpep* intervention and completed the post-treatment assessment. No control condition was included in this study.

### 2.2 Recruitment

All students enrolled at the participating universities received a link to an online survey via their student email addresses. This survey included questions on demographics, various aspects of mental health -such as depression and stress symptomatology- and quality of life. After finishing the survey, students were advised to participate in the intervention(s) that matched their screening results. They were free to choose the intervention they preferred but could only participate in one intervention at a time. Possible interventions were the Moodpep intervention, which aimed to reduce depression symptomatology (i.e., the focus of the current study), as well as interventions targeting relaxation, life skills or sleep, or to decrease procrastination or distress due to the COVID-19 pandemic. Care providers within the universities, such as psychologists, could also refer students directly to specific interventions, including the *Moodpep* intervention.

### 2.3 Eligibility criteria

To be eligible for the current study, participants had to meet the following inclusion criteria: (a) experiencing at least mild depression symptomatology (PHQ-9 score ≥ 5), (b) being enrolled as a student or affiliated as a PhD student at one of the nine participating universities, (c) having internet access through a computer or mobile device, (d) fluency in Dutch or English, (e) being 16 years or older (Garnefski and Kraaij, 2023). At the start of the study, students with current suicidal ideation, past suicide attempts and/or severe depression symptomatology (PHQ-9 score ≥ 20) were excluded from the study and advised to seek professional help. However, current suicidal ideation was removed as an exclusion criterion in October 2022, and severe depression was removed in March 2023.

### 2.4 Samples

**Fig. 1:**
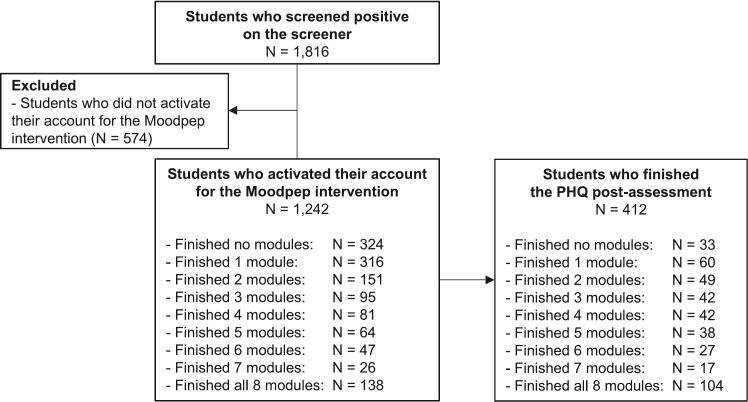
Flow chart of the study.

For the first set of research aims (i.e., aims 1.1–1.3), we report pre-treatment findings from all 1816 participants who were eligible for the *Moodpep* intervention during three years of screening (June 2020–June 2023). Of the 1816 students who were eligible for the *Moodpep* intervention, 1242 (68.4 %) activated their account for the intervention (see Fig. 1 for the flow chart). Of those students, 412 (33.3 %) completed the post-treatment assessment and comprised the sample for our second set of research aims (i.e., aims 2.1–2.3). The low uptake is most likely related to the Caring Universities platform infrastructure, with very low-threshold access to the interventions and limited guidance in the onboarding process. It is however important to note that these numbers are comparable to studies with a similar design; e.g., of the 8014 youth that signed up for a digital intervention that was integrated within Australian youth mental health services, <20 % filled in the pre- and post-treatment assessment (Alvarez-Jimenez et al., 2025).

### 2.5 Intervention

The *Moodpep* intervention was introduced to students as an online self-help program supplemented with coaching for those experiencing feelings of depression (for full details on the intervention, please, see Garnefski and Kraaij, 2023). It was specifically designed for students experiencing mild to moderate depression symptomatology and comprised eight weekly modules, each requiring approximately one to two hours to complete. The modules consisted of psychoeducation and assignments and focussed on Cognitive Behavioural Therapy techniques. They were structured around four components: behavioural activation, relaxation exercises, changing negative thoughts and goal setting. Participants followed the modules online via the Caring Universities platform. While it was recommended to complete one module each week, participants had the flexibility to work at their own pace. After every module, a coach gave written feedback on the assignments through a chat tool integrated into the online platform. This is slightly different from the telephone coaching that was used in the pilot study on the intervention (Garnefski and Kraaij, 2023). The pilot study was conducted at the clinical facility of Leiden University and included only 32 participants, whereas the current study operates on a larger scale via the Caring Universities platform and has the potential to reach thousands of participants. Providing support through written feedback, integrated in the platform, makes the intervention more efficient and easier to scale.

### 2.6 Measures

Key measures of the current study included depression and stress symptomatology as well as overall quality of life, all assessed at baseline and directly after the *Moodpep* intervention.

#### 2.6.1 Depression symptomatology

Depression symptomatology was assessed with the Patient Health Questionnaire (PHQ-9; Kroenke et al., 2001), a 9-item self-report questionnaire measuring all nine DSM-based symptoms of a major depressive disorder. The nine items assess the frequency of symptoms during the past two weeks, with scores ranging from 0 (never) to 3 (almost every day). We considered the sum score of all nine symptoms as a measure of overall depression symptomatology (potential range: 0–27; Cronbach's alpha: 0.80), while scores on all individual items were also considered to measure each of the nine individual depression symptoms. It is important to note that the reliability and validity of assessing individual depression symptoms with individual items of the PHQ-9 are unknown.

#### 2.6.2 Stress symptomatology

Stress symptomatology was assessed with the Perceived Stress Scale (PSS-10; Cohen et al., 1983), a 10-item self-report questionnaire measuring perceived stress in daily life. The questionnaire comprises ten items covering the frequency of feelings of helplessness (items 1, 2, 3, 6, 9 and 10) and lack of self-efficacy (items 4, 5, 7 and 8) experienced in the past month, with responses ranging from 0 (never) to 4 (very often). We considered the sum score of all ten items as a measure of overall stress symptomatology (potential range: 0–40; Cronbach's alpha: 0.79) as well as the scores on each of the ten separate items as measures of all ten individual stress symptoms. It is important to note that the reliability and validity of assessing individual stress symptoms with individual items of the PSS-10 are unknown.

#### 2.6.3 Overall quality of life

Overall quality of life was assessed with a single item from the Mental Health Quality of Life questionnaire that has been developed for people with mental health problems (MHQoL; Van Krugten et al., 2021). The MHQoL is an 8-item self-report questionnaire, with seven items addressing specific dimensions of quality of life (i.e., self-image, independence, mood, relationships, daily activities, physical health, future), along with one item on overall psychological well-being. This last item asks students to indicate their overall psychological well-being on the day of the assessment, with scores ranging from 0 (worst imaginable psychological well-being) to 10 (best imaginable psychological well-being). We specifically focussed on this last item, as we wanted to examine how (changes in) specific depression and stress symptoms were related to (changes in) overall quality of life. As specific symptoms of depression show large overlap with specific dimensions of quality of life (e.g., depression symptom ‘feeling depressed/hopeless’ and quality of life dimension ‘mood’, depression symptom ‘feeling guilty/worthless’ and quality of life dimension ‘self-image’), isolating this single item helped prevent potential distortion in our findings. However, it is important to note that the reliability and validity of assessing overall quality of life with this single item are unknown.

### 2.7 Data analysis

All analyses were performed using SPSS version 29.0.2.0, except for the network estimations which were performed using R version 4.3.1. We reported the baseline characteristics (i.e., demographics, overall depression and stress symptomatology as well as overall quality of life) in the 1816 students were eligible for the *Moodpep* intervention (i.e., sample for research aims 1.1 to 1.3) and the 412 students who activated their account for the *Moodpep* intervention and completed the post-treatment assessment (i.e., sample for research aims 2.1 to 2.3).

#### 2.7.1 Research aims 1.1 to 1.3

To determine the frequencies of individual symptoms (aim 1.1), we reported the mean and standard deviations of individual depression and stress symptoms at the pre-treatment assessment. In the next step, we focused on the interrelations among these symptoms (aim 1.2) as well as their relations with overall quality of life (aim 1.3). Therefore, a network including all nine depression symptoms, all ten stress symptoms as well as the overall quality of life score was estimated using L1-regularized partial correlations (Friedman et al., 2010; gamma = 0.25) and visualized using package *qgraph* (Epskamp et al., 2012). We first focused on the depression symptoms and how they were related to other depression and stress symptoms. For each of these depression symptoms, we calculated the sum of connections with other symptoms, as a measure for the number and/or strength of connections with other symptoms. Next, we evaluated the connection strengths of depression and stress symptoms with overall quality of life.

#### 2.7.2 Research aims 2.1 to 2.3

To determine whether overall depression symptomatology, overall stress symptomatology and overall quality of life changed during the *Moodpep* intervention, paired-samples *t*-tests were performed to compare post-treatment and pre-treatment scale scores; effect sizes (Cohen's *d*) were calculated. Similar analyses were used to determine whether individual depression and stress symptoms changed during the *Moodpep* intervention (aim 2.1). In the next step, we estimated a network including changes in all nine depression symptoms, changes in all ten stress symptoms, and the change in overall quality of life score; the methodology mirrored that of the previous network (see the analyses for research aim 1.2 and 1.3). We first focused on the changes in depression symptoms and how they were related to changes in other depression and stress symptoms (aim 2.2) and, again, calculated the sum of connections for each of the depression symptoms. Secondly, we considered the connection strengths of changes in depression and stress symptoms with change in overall quality of life (aim 2.3).

As a set of exploratory analyses, we explored whether the number of completed *Moodpep* modules (three categories: 0–2 modules, 3–6 modules and 7–8 modules) was related to changes in depression and stress symptomatology as well as changes in overall quality of life. First, multiple analyses of variance (ANOVAs) were performed to test whether changes in overall depression symptomatology, overall stress symptomatology, overall quality of life as well as all nine depression and all ten stress symptoms differed across groups based on the number of completed modules. Second, the R package *Network Comparison Test* (Van Borkulo et al., 2023) was used to test potential differences in networks including changes in individual depression symptoms, individual stress symptoms and overall quality of life differed across the three module completion groups.

## 3 Results

### 3.1 Sample characteristics

In total, 1816 students were eligible to participate in the *Moodpep* intervention and comprised the sample for our analyses on pre-treatment scores (aims 1.1 to 1.3). Of those, 412 students activated their account for the intervention and completed the post-treatment assessment, thus comprising the sample for our analyses on change scores (aims 2.1 to 2.3). Table 1 shows the demographic characteristics and baseline information about overall depression and stress symptomatology as well as overall quality of life for both samples.

**Table 1:** Sample characteristics.

	Sample for research aim 1^a^ (*N* = 1816)		Sample for research aim 2^b^ (*N* = 412)	
	N	Mean (*SD*) / *N* (%)	N	Mean (*SD*) / *N* (%)
Age, in years	1816	22.8 (4.3)	412	22.9 (4.1)
Gender	1816		412	
Female		1441 (79.4 %)		331 (80.3 %)
Male		359 (19.8 %)		80 (19.4 %)
Other		16 (0.9 %)		1 (0.2 %)
Education level	1816		412	
Bachelor		1001 (55.1 %)		205 (49.8 %)
Master		757 (41.7 %)		186 (45.1 %)
PhD		58 (3.2 %)		21 (5.1 %)
Marital status	1816		412	
Single		1137 (62.6 %)		249 (60.4 %)
In a relationship / Married		616 (33.9 %)		150 (36.4 %)
Married		36 (2.0 %)		9 (2.2 %)
Divorced		5 (0.3 %)		2 (0.5 %)
Other		22 (1.2 %)		2 (0.5 %)
Nationality	1816		412	
Dutch		956 (52.6 %)		224 (54.4 %)
European (non-Dutch)		643 (35.4 %)		152 (36.9 %)
Other		217 (11.9 %)		36 (8.7 %)
Treatment	1816		412	
None		1477 (81.3 %)		348 (84.5 %)
Psychological counselling		186 (10.2 %)		43 (10.4 %)
Medication		99 (5.5 %)		17 (4.1 %)
Both		54 (3.0 %)		4 (1.0 %)
Overall depression symptomatology (PHQ-9 sum score)	1816	12.7 (5.1)	412	11.1 (4.1)
Overall stress symptomatology (PSS-10 sum score)	1744	23.8 (5.6)	412	22.6 (5.4)
Overall quality of life (MHQoL item 8)	1510	4.2 (1.6)	354	4.5 (1.5)

a Students who screened positive on the screener.

b Students who registered for the *Moodpep* intervention and completed the PHQ-9 post-assessment.

### 3.2 Depression and stress symptoms, quality of life and their interrelations (aims 1.1 to 1.3)

**Fig. 2:**
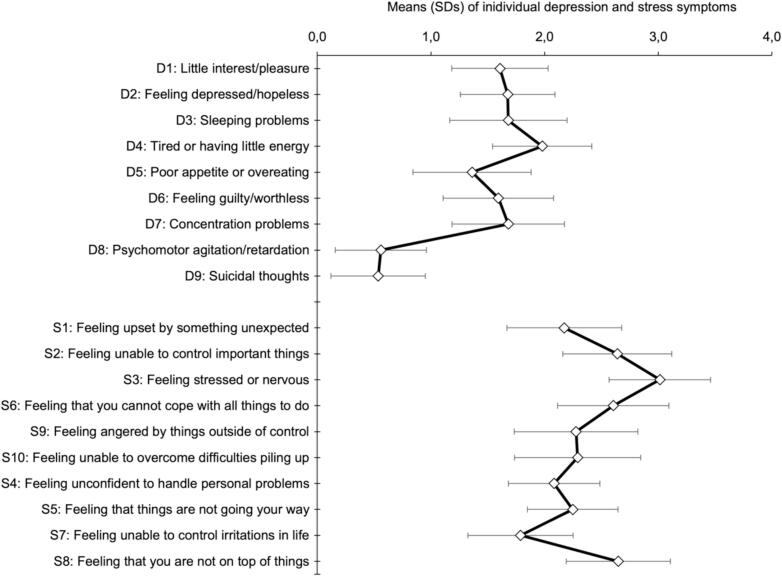
Mean and standard deviations (SDs) of individual depression symptoms (range 0–3) and individual stress symptoms (range 0–4).

Fig. 2 shows the descriptive statistics of all individual depression and stress symptoms in our sample of 1816 student who were eligible to participate in the *Moodpep* intervention (aim 1.1). Mean scores on individual depression symptoms varied substantially, with the highest mean score observed for ‘feeling tired or having little energy’ (D4; mean = 2.0, SD = 0.9) and the lowest for ‘suicidal thoughts’ (D9; mean = 0.5, SD = 0.8). Also, mean scores on individual stress symptoms varied substantially, with ‘feeling stressed or nervous’ scoring the highest (S3; mean = 3.0, SD = 0.9) and ‘feeling unable to control irritations in life’ the lowest (S7; mean = 1.8, SD = 0.9).

**Fig. 3:**
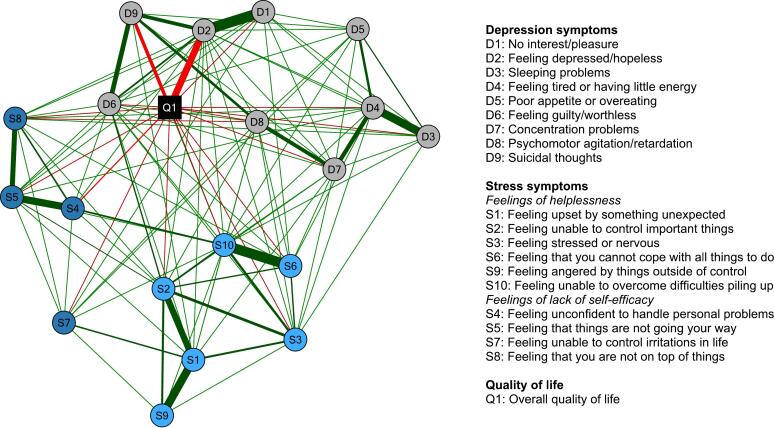
Network structure of individual depression and stress symptoms as well as overall quality of life in our sample of students eligible to participate in the *Moodpep* intervention (*N* = 1446).

To determine the interrelations between these depression and stress symptoms (aim 1.2) and their relations with overall quality of life (aim 1.3), a network was estimated (Fig. 3; see supplemental Table S1 for the exact edge strengths). Depression symptoms were highly connected to each other (i.e., with 30 of 36 possible connections, 83.3 %), with ‘feeling depressed/hopeless’ (D2; sum of connections: 0.80) and ‘feeling tired or having little energy (D4; sum of connections: .75) showing the highest sum of connections with other depression symptoms. Depression symptoms also showed a high number of connections to stress symptoms (i.e., with 42 of 90 possible connections, 46.7%; 28 with feelings of helplessness and 14 with lack of self-efficacy items), with ‘feeling guilty/worthless' (D6; sum of connections: .34) showing the highest sum of connections with stress symptoms. In total, eight (of nine) depression symptoms and eight (of ten) stress symptoms were (negatively) related to quality of life (sum of connections: -.69 and -.47, respectively). ‘Feeling depressed/hopeless' (D2; connection strength: −0.22) and ‘suicidal thoughts' (D9; connection strength: −0.15) showed the strongest connections of all depression symptoms with overall quality of life, and ‘feeling unconfident to handle personal problems' (S4; connection strength: −0.10) and ‘feeling unable to overcome difficulties piling up’ (S10; connection strength: −0.09) of all stress symptoms.

### 3.3 Changes in depression and stress symptoms, quality of life and their interrelations after the intervention (aims 2.1 to 2.3)

In the next step, we zoomed in on the 412 students who activated their account for the *Moodpep* intervention and completed the post-treatment assessment. Overall depression symptomatology and overall stress symptomatology showed medium reductions after the intervention (Cohen's *d*: 0.573 and 0.572, respectively; both *p* < .001), whereas overall quality of life showed a strong increase (Cohen's *d*: −0.825; p < .001).

**Fig. 4:**
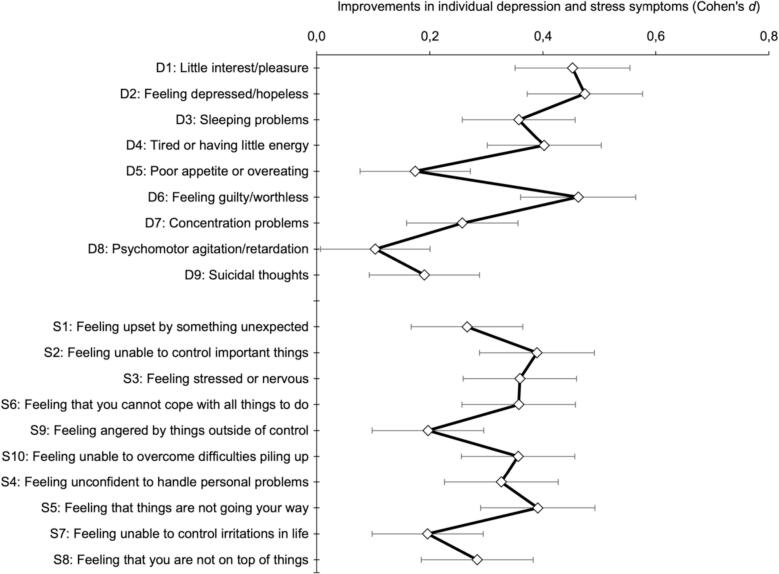
Improvements in individual depression symptoms and stress symptoms (Cohen's *d*, with 95 % confidence intervals).

Fig. 4 shows the changes in individual depression and stress symptoms after the intervention (aim 2.1). All individual symptoms showed significant reductions (all *p*-values <.001, except for ‘psychomotor agitation/retardation’ *p* = .04), but substantial differences in effects sizes were found across symptoms. Of the nine depression symptoms, the largest reductions were observed for ‘feeling depressed/hopeless’, ‘feeling guilty/worthless’ and ‘little interest/pleasure’ (D2, D6, D1; Cohen's *d*: 0.47, 0.46 and 0.45, respectively), whereas the smallest reductions were found for ‘psychomotor agitation/retardation’ and ‘poor appetite or overeating’ (D8, D5; Cohen's *d*: 0.10 and 0.17, respectively). Of the ten stress symptoms, ‘feeling that things are not going your way’ and ‘feeling unable to control important things’ showed the largest reductions (S5, S2; Cohen's *d*: 0.39, both), while ‘feeling unable to control irritations in life’ and ‘feeling angered by things outside of control’ showed the smallest (S7, S9; Cohen's *d*: 0.20, both).

**Fig. 5:**
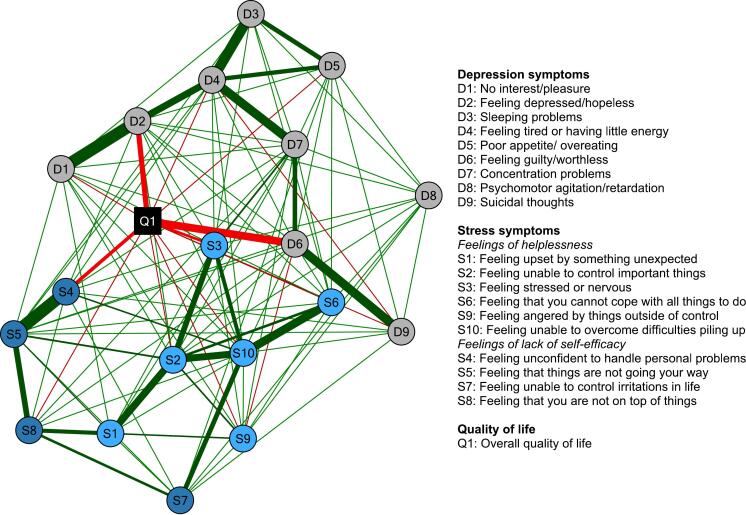
Network structure of changes of individual depression and stress symptoms as well as overall quality of life in our sample of students who registered for the Moodpep intervention had valid data at the post-treatment assessment (*N* = 351).

In a next step, we estimated a network to determine the interrelations among changes in depression and stress symptoms (aim 2.2) and their relations with quality of life (aim 2.3) (Fig. 5; see supplemental Table S2 for the exact strengths). Changes in depression symptoms were highly connected to each other (i.e., with 25 of 36 possible connections, 69.4 %), with ‘feeling tired or having little energy (D4; sum of connections: .83) and ‘feeling depressed/hopeless' (D2; sum of connections: .66) having the highest sum of connections with other depression symptoms. Changes in depression symptoms also showed a high number of connections to changes in stress symptoms (i.e., with 42 of 90 possible connections, 46.7%; 27 feelings of helplessness and 15 with lack of self-efficacy items), with ‘concentration problems' (D7; sum of connections: 0.28) having the highest sum of connections with changes in stress symptoms and ‘poor appetite or overeating’ (D5; cum of connections: 0.10) the lowest. In total, changes in five (of nine) depression symptoms and eight (of ten) stress symptoms were (negatively) related to changes in quality of life (sum of connections: −0.52 and − 0.50, respectively). Changes in ‘feeling tired or having little energy’ (D6; connection strength: −0.19) and ‘feeling depressed/hopeless' (D2; connection strength: −0.16) had the strongest connections of all depression symptoms with changes in quality of life, and changes in ‘feeling unconfident to handle personal problems' (S4; connection strength: −0.13) and ‘feeling stressed or nervous' (S3; connection strength: −0.12) of all stress symptoms.

As part of a set of exploratory analyses, we first explored whether the number of finished *Moodpep* modules was related to changes in depression and stress symptomatology and changes in overall quality of life. We found no significant differences in changes in overall depression symptomatology (*p* = .14), overall stress symptomatology (*p* = 1.00) and overall quality of life (*p* = .28) between students who finished 0–2 modules, 3–6 modules and 7–8 modules. In addition, no significant differences were found for any of the individual depression and stress symptoms, except the depression symptoms ‘poor appetite/overeating’ (D5; mean scores: −0.21, −10 and − 0.23, respectively) and ‘psychomotor agitation/retardation’ (D8; mean scores: −0.09, −0.09, −0.02, respectively). We also examined whether the interrelations of individual depression symptoms, individual stress symptoms and overall quality of life differed across these three groups based on the number of finished modules. No significant differences were found between the three networks (network invariance tests: M = 0.25, *p* = .48 for 0–2 versus 3–6 modules; M = 0.27, *p* = .25 for 3–6 versus 7–8 modules; M = 0.27, *p* = .39 for 0–2 versus 7–8 modules).

## 4 Discussion

### 4.1 Principal findings

The current study is the first to examine symptom heterogeneity in students experiencing mild to severe depression symptomatology (research aims 1.1 to 1.3; *N* = 1816) as well as symptom-specific changes after a guided iCBT intervention (Moodpep) that was tailored specifically to students (research aims 2.1 to 2.3; *N* = 412). We found that mean scores of individual depression and stress symptoms differed substantially in our sample and that they were strongly connected to each other. Among the depression symptoms, ‘feeling depressed/hopeless’ and ‘suicidal thoughts’ showed the strongest (negative) connections with overall quality of life, stressing their relevance in students' daily lives. After the intervention, we observed medium reductions in overall depression symptomatology and stress symptomatology, whereas overall quality of life showed a strong increase. Although all individual depression and stress symptoms decreased, effect sizes differed substantially, and changes in these symptoms were strongly interrelated. Changes in ‘feeling tired or having little energy’ and ‘feeling depressed/hopeless’ showed the strongest (negative) connections with overall quality of life, underlining their clinical relevance.

### 4.2 Symptom heterogeneity in students

The scores of individual depression symptoms differed substantially in our large sample of students experiencing mild to severe depression symptomatology. ‘Feeling tired or having little energy’ was the most frequently reported symptom, directly followed by most other symptoms, except for ‘psychomotor agitation/retardation’ and ‘suicidal thoughts’ which were less commonly reported. These patterns align with findings from a study in adults with mild to moderate depression symptomatology (Boschloo et al., 2019a) and -although based on a different measure and therefore not fully comparable- with research involving a large sample of college students (Mullarkey et al., 2018). Interestingly, the interrelations between depression symptoms were also highly similar across samples; for example, ‘feeling depressed/hopeless’ and ‘feeling tired and having little energy’ have the strongest connections with other depression symptoms in our sample and in the sample of adults with mild to moderate symptomatology (Boschloo et al., 2019a). Collectively, this underscores the robustness and replicability of the observed patterns across diverse populations experiencing depression symptomatology.

Our study also provides important additional information, such as the significant prevalence of perceived stress symptoms among students and their strong interrelatedness with depression symptoms. In addition, our students reported substantial impairments in quality of life (i.e., mean score of 4.2 on a scale of 0–10, with 10 representing the best imaginable psychological well-being). ‘Feeling depressed/hopeless’ and ‘suicidal thoughts’ showed the strongest negative associations with quality of life, indicating that especially these symptoms are burdensome in students' lives. This is partly in line with a study on the relative importance of individual symptoms on impairment in adults with a major depressive disorder (Fried and Nesse, 2014). Together, our findings suggest that ‘feeling depressed/hopeless’ and ‘suicidal thoughts’ would be valuable targets for intervention strategies.

### 4.3 Symptom-specific changes after the iCBT intervention

The current study is also an important follow-up of the pilot study of Garnefski and Kraaij (2023), who reported a large reduction in overall depression symptomatology (Cohen's *d* = 0.94) in students participating in the *Moodpep* intervention (with telephone coaching). Our study included a much larger sample and showed that this effect size was a bit smaller (Cohen's *d* = 0.57) for the Moodpep intervention (with online coaching). Our most important finding is, however, that individual symptoms differed substantially in their reductions after the intervention (Cohen's *d*: 0.10–0.47), which is in line with previous studies including the one on differential symptom-specific effects of an iCBT intervention for adults with mild to moderate depression symptomatology relative to a care as usual condition (Boschloo et al., 2019a). Especially ‘feeling depressed/hopeless’, ‘feeling guilty/worthless’, ‘little interest/pleasure’ and ‘feeling tired or having little energy’ showed considerable reductions after the intervention. This could potentially be explained by the focus of the intervention on, for example, changing negative thoughts and behavioural activation (increasing activities that one enjoys). Smaller effect sizes were observed for symptoms that were not directly addressed by the intervention, such as ‘psychomotor agitation/retardation’ and ‘poor appetite or overeating’.

Our study further showed that changes in individual depression symptoms after the intervention were strongly related to changes in other depression symptoms as well as stress symptoms including symptoms of feelings of helplessness and, to a lesser extent, lack of self-efficacy. Perceived helplessness is the belief that a situation is beyond one's control (Klein et al., 1976) and a lack of self-efficacy refers to a lack of confidence in the ability to achieve goals (Bandura, 1977) and both are often seen in individuals with depression due to their negative cognitions and self-evaluations. It is, therefore, not surprising that especially ‘feeling guilty/worthless’, ‘feeling depressed/hopeless’ and ‘concentration problems’ were most strongly related to stress symptoms in the networks that included either baseline scores or change scores for individual symptoms. In line with previous trials (Boschloo et al., 2022, Boschloo et al., 2023), these findings underscore the importance of not only considering key symptoms of depression in research on treatment efficacy but also considering other mental health indicators for a more complete and realistic measure of treatment response.

Moreover, our findings indicated that reductions in depression symptoms and, to a lesser extent, stress symptoms were related to increases in overall quality of life. Especially reductions in ‘feelings of guilt/worthlessness’, ‘feeling depressed/hopeless’, ‘little interest/pleasure’ and “feeling tired or having little energy’ were most strongly related to increases in overall quality of life, underlining their clinical relevance. Interestingly, these symptoms also showed the strongest reductions after the *Moodpep* intervention. This may imply that especially students with these specific depression symptoms will benefit from this intervention, not only due to the larger reductions in these depression symptoms (relative to other depression symptoms) but also due to their related increases in quality of life.

In future research, it would be interesting to also consider other types of interventions for students with depression symptomatology and to determine the symptom-specific effects of such interventions relative to a control condition or to other interventions. Students primarily suffering from symptoms that are directly targeted by an intervention benefit -by definition- more from that intervention than students primarily suffering from other symptoms. Information about the symptom-specific effects of interventions could therefore improve the identification of students who -based on their exact pre-treatment symptomatology- would benefit the most from that intervention. Such an approach has already shown to be valuable in previous studies (see, for example, Boschloo et al., 2019a, Boschloo et al., 2019b, Boschloo et al., 2022). For example, our study on the symptom-specific effects of an iCBT intervention compared to care usual (Boschloo et al., 2019a) showed that a pre-treatment severity measure -based on the four symptoms that were directly targeted by that intervention- moderated the effect of this intervention on overall depression symptomatology; that is, the effect size ranged from 0.15 (not significant) for those scoring the lowest compared to 0.50 (significant) for those scoring the highest on this measure. Such a symptom-specific approach can, thus, guide informed treatment recommendations and would be a valuable first step towards personalized medicine.

### 4.4 Strengths, limitations and suggestions for further research

Strengths of the current study lie in its large sample size and its detailed examination of individual depression and stress symptoms as well as their interrelations. This comprehensive approach allows for a more detailed and nuanced assessment of symptom heterogeneity (*N* = 1816) and offers valuable insights into the process of change during the intervention (*N* = 412). In addition, we were the first to consider quality of life and explore which (changes in) symptoms were related to (changes in) quality of life. However, it would be valuable to also incorporate other outcomes that are strongly related to depression symptomatology, such as anxiety and other psychiatric symptoms, along with educational underachievement and drop-out (Beard et al., 2016; Jenkins et al., 2021).

In addition, it is important to note that the reliability and validity of assessing individual symptoms with individual items of, for example, the PHQ-9 (Kroenke et al., 2001) and the PSS-10 (Cohen et al., 1983) are largely unknown. The same holds true for assessing overall quality of life with a single item of the MHQoL (Van Krugten et al., 2021). Furthermore, a specific limitation of the PHQ-9 is that it aggregates two sets of opposite symptoms, i.e., poor appetite and overeating, and psychomotor agitation and retardation. Previous research has shown that disaggregated symptoms exhibit differential symptom scores as well as differential connections with symptoms (Boschloo et al., 2015) and, therefore, aggregation of symptoms may have distorted our findings and could, for example, explain the small reductions in these two symptoms after the intervention, as well as their weak connections with changes in other symptoms and quality of life. It would be highly interesting to consider disaggregated symptoms in future research on treatment efficacy.

It is also important to note that depression and stress symptoms with higher pre-treatment scores had more room for improvement and, indeed, showed larger effect sizes after the intervention (i.e., correlation of the mean pre-treatment score with the effect size after the intervention across symptoms: *r* = 0.76 for depression symptoms and *r* = 0.47 for stress symptoms). In addition, our study did not include a control condition, so it is unclear whether reductions in symptoms are a consequence of the *Moodpep* intervention, or from other factors, such as the naturalistic course of symptoms and/or the impact of the coaching or other psychological or pharmacological treatments. In this light, it is remarkable that the number of finished modules is not related to changes in overall symptomatology and most individual symptoms. This may imply that the intervention is not efficacious in reducing symptomatology. However, it is also possible that students who benefit from the intervention, also in the first weeks, stop participating. Future research should therefore include a control condition to more stringently determine the efficacy of the intervention and to take into account potential dropouts. An intention-to-treat analysis -instead of analyses in completers as in the current study- would be preferable, as this preserves the group randomization, has greater generalizability, maintains the sample size, and reduces bias.

Another substantial limitation is that conclusions regarding the direction of associations between changes in symptoms in the network are precluded, as symptoms were only assessed immediately before and after the intervention. Assessing symptom scores multiple times throughout the intervention, for example after each module, could be beneficial in determining the causal relations between symptoms as well as the potential impact of the individual modules on these symptoms. This approach would shed light on the mechanisms underlying the interventions and their separate modules, providing valuable information about symptoms that, while not directly targeted by the intervention, could be the focus of other interventions. Such insights may lead to the development of more effective and tailored iCBT interventions for students experiencing mild to severe depression symptomatology.

### 4.5 Conclusion

The current study examined symptom heterogeneity in students experiencing mild to severe depression symptomatology as well as symptom-specific changes after a guided iCBT intervention (*Moodpep*) that was specifically tailored to students. Our findings highlight the importance of considering (changes in) individual symptoms and their interrelations as a more complete and nuanced measure of symptomatology in students, as well as a more complete and nuanced measure of their process of change during an intervention. This approach has the potential to guide clinicians towards more precisely informed treatment recommendations and could even lead to more effective interventions for depression symptomatology.

## Declaration of competing interest

The authors declare that they have no known competing financial interests or personal relationships that could have appeared to influence the work reported in this paper.

## Appendix A Supplementary data

Supplementary tables Image 1
