# Supplementary material for: Symptom heterogeneity in students with mild to severe depression symptomatology and their differential symptom-specific changes during an internet-based, guided cognitive behavioural therapy intervention
Source: Internet Interv. 2025 May 16;41:100834. doi: 10.1016/j.invent.2025.100834 (PMC12155852; doi:10.1016/j.invent.2025.100834)
Supplement: Supplementary file 1 — Supplementary tables [file mmc1.docx]

**TABLES AND FIGURES**

**Figure 1. Flow chart of the study.**

**Table 1. Sample characteristics.**

|  | Sample for  research aim 1^a^  (N=1,816) | | Sample for  research aim 2^b^  (N=412) | |
| --- | --- | --- | --- | --- |
|  | **N** | **mean (*SD*) / *N* (%)** | **N** | **Mean (*SD*) / *N* (%)** |
| Age, in years | 1,816 | 22.8 (4.3) | 412 | 22.9 (4.1) |
| Gender | 1,816 |  | 412 |  |
| Female |  | 1,441 (79.4%) |  | 331 (80.3%) |
| Male |  | 359 (19.8%) |  | 80 (19.4%) |
| Other |  | 16 (0.9%) |  | 1 (0.2%) |
| Education level | 1,816 |  | 412 |  |
| Bachelor |  | 1,001 (55.1%) |  | 205 (49.8%) |
| Master |  | 757 (41.7%) |  | 186 (45.1%) |
| PhD |  | 58 (3.2%) |  | 21 (5.1%) |
| Marital status | 1,816 |  | 412 |  |
| Single |  | 1,137 (62.6%) |  | 249 (60.4%) |
| In a relationship / Married |  | 616 (33.9%) |  | 150 (36.4%) |
| Married |  | 36 (2.0%) |  | 9 (2.2%) |
| Divorced |  | 5 (0.3%) |  | 2 (0.5%) |
| Other |  | 22 (1.2%) |  | 2 (0.5%) |
| Nationality | 1,816 |  | 412 |  |
| Dutch |  | 956 (52.6%) |  | 224 (54.4%) |
| European (non-Dutch) |  | 643 (35.4%) |  | 152 (36.9%) |
| Other |  | 217 (11.9%) |  | 36 (8.7%) |
| Treatment | 1,816 |  | 412 |  |
| None |  | 1,477 (81.3%) |  | 348 (84.5%) |
| Psychological counselling |  | 186 (10.2%) |  | 43 (10.4%) |
| Medication |  | 99 (5.5%) |  | 17 (4.1%) |
| Both |  | 54 (3.0%) |  | 4 (1.0%) |
| Overall depression symptomatology (PHQ-9 sum score) | 1,816 | 12.7 (5.1) | 412 | 11.1 (4.1) |
| Overall stress symptomatology (PSS-10 sum score) | 1,744 | 23.8 (5.6) | 412 | 22.6 (5.4) |
| Overall quality of life (MHQoL item 8) | 1,510 | 4.2 (1.6) | 354 | 4.5 (1.5) |

^a^ Students who screened positive on the screener

^b^ Students who registered for the *Moodpep* intervention and completed the PHQ-9 post-assessment

**Figure 2. Mean and standard deviations (SDs) of individual depression symptoms (range 0-3) and individual stress symptoms (range 0-4).**

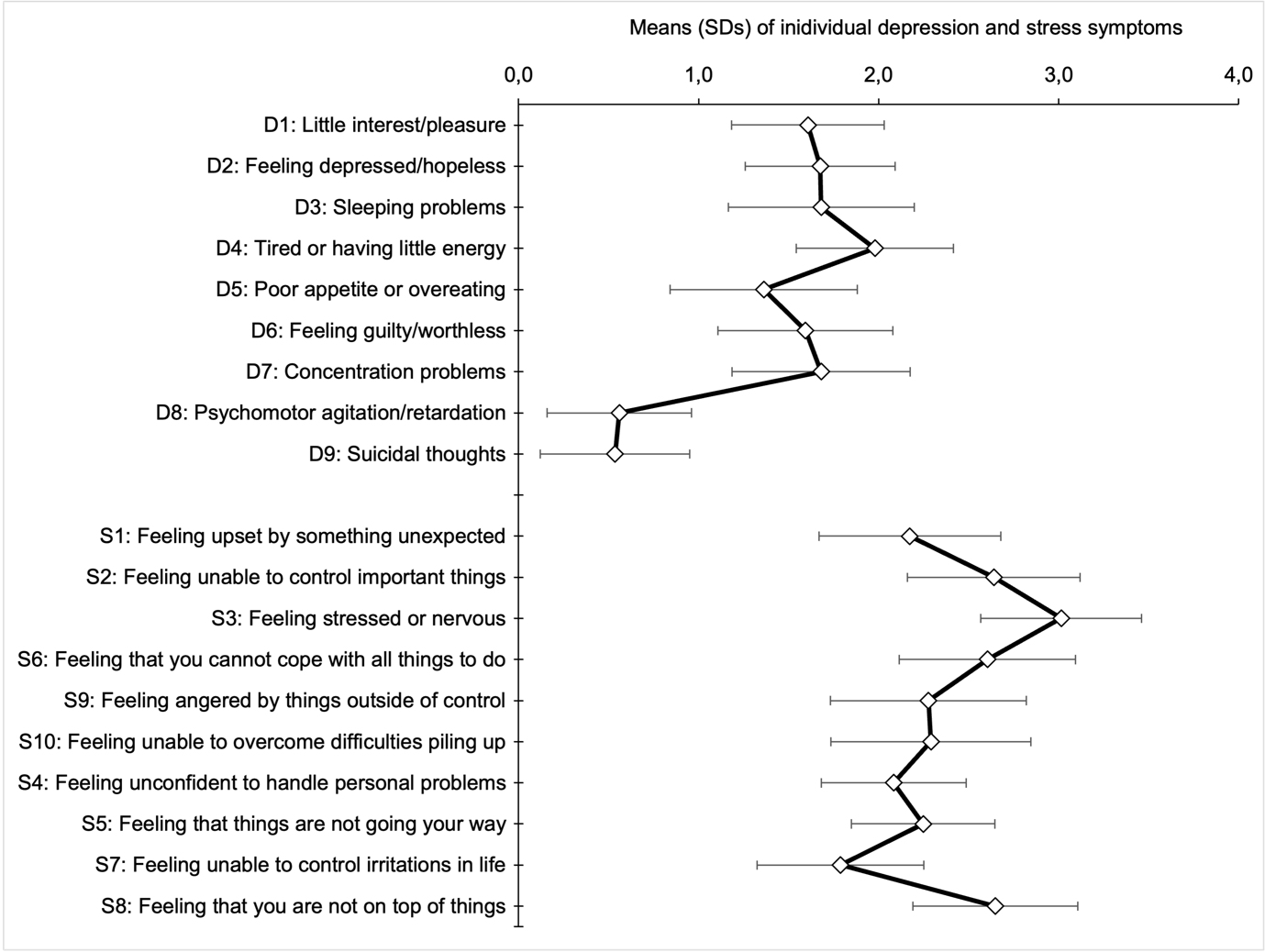


**Figure 3. Network structure of individual depression and stress symptoms as well as overall quality of life in our sample of students eligible to participate in the *Moodpep* intervention (N=1,446).**

| **** | **Depression symptoms**  D1: No interest/pleasure  D2: Feeling depressed/hopeless  D3: Sleeping problems  D4: Feeling tired or having little energy  D5: Poor appetite or overeating  D6: Feeling guilty/worthless  D7: Concentration problems  D8: Psychomotor agitation/retardation  D9: Suicidal thoughts  **Stress symptoms**  *Feelings of helplessness*  S1: Feeling upset by something unexpected  S2: Feeling unable to control important things  S3: Feeling stressed or nervous  S6: Feeling that you cannot cope with all things to do  S9: Feeling angered by things outside of control  S10: Feeling unable to overcome difficulties piling up  *Feelings of lack of self-efficacy*  S4: Feeling unconfident to handle personal problems  S5: Feeling that things are not going your way  S7: Feeling unable to control irritations in life  S8: Feeling that you are not on top of things  **Quality of life**  Q1: Overall quality of life |
| --- | --- |

**Figure 4. Improvements in individual depression symptoms and stress symptoms (Cohen’s *d*, with 95% confidence intervals).**


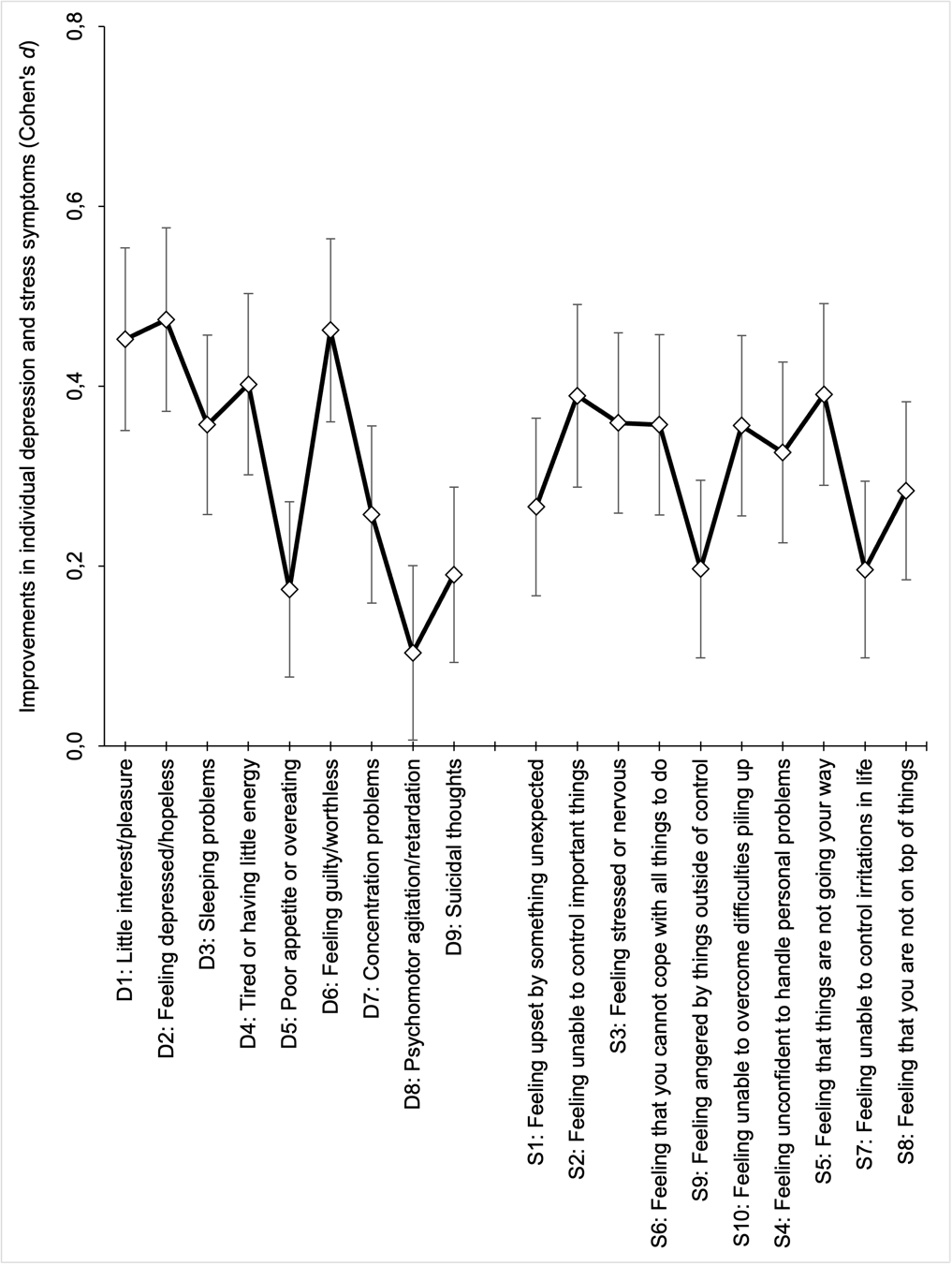


**Figure 5. Network structure of changes of individual depression and stress symptoms as well as overall quality of life in our sample of students who registered for the Moodpep intervention had valid data at the post-treatment assessment (N=351).**

| **** | **Depression symptoms**  D1: No interest/pleasure  D2: Feeling depressed/hopeless  D3: Sleeping problems  D4: Feeling tired or having little energy  D5: Poor appetite/ overeating  D6: Feeling guilty/worthless  D7: Concentration problems  D8: Psychomotor agitation/retardation  D9: Suicidal thoughts  **Stress symptoms**  *Feelings of helplessness*  S1: Feeling upset by something unexpected  S2: Feeling unable to control important things  S3: Feeling stressed or nervous  S6: Feeling that you cannot cope with all things to do  S9: Feeling angered by things outside of control  S10: Feeling unable to overcome difficulties piling up  *Feelings of lack of self-efficacy*  S4: Feeling unconfident to handle personal problems  S5: Feeling that things are not going your way  S7: Feeling unable to control irritations in life  S8: Feeling that you are not on top of things  **Quality of life**  Q1: Overall quality of life |
| --- | --- |

**Supplemental Table S1. Connection strengths between individual depression and stress symptoms as well as overall quality of life (underlying Figure 3).**

|  | **D1** | **D2** | **D3** | **D4** | **D5** | **D6** | **D7** | **D8** | **D9** | **S1** | **S2** | **S3** | **S6** | **S9** | **S10** | **S4** | **S5** | **S7** | **S8** | **Q1** |
| --- | --- | --- | --- | --- | --- | --- | --- | --- | --- | --- | --- | --- | --- | --- | --- | --- | --- | --- | --- | --- |
| **D1** | - | 0,30 | 0,06 | 0,09 | 0,10 | 0,03 | 0,08 | 0,00 | 0,00 | 0,00 | 0,00 | 0,00 | 0,01 | 0,00 | 0,00 | 0,00 | 0,04 | 0,02 | 0,04 | -0,08 |
| **D2** | 0,30 | - | 0,04 | 0,08 | 0,05 | 0,12 | 0,03 | 0,03 | 0,16 | 0,01 | 0,04 | 0,01 | 0,00 | 0,00 | 0,04 | 0,04 | 0,03 | 0,00 | 0,02 | -0,22 |
| **D3** | 0,06 | 0,04 | - | 0,25 | 0,10 | 0,00 | 0,01 | 0,05 | 0,00 | 0,00 | 0,00 | 0,01 | 0,00 | 0,00 | 0,04 | 0,00 | 0,00 | 0,00 | 0,01 | -0,03 |
| **D4** | 0,09 | 0,08 | 0,25 | - | 0,13 | 0,00 | 0,17 | 0,02 | 0,00 | 0,00 | 0,00 | 0,05 | 0,04 | 0,00 | 0,03 | 0,00 | 0,00 | 0,00 | 0,02 | -0,04 |
| **D5** | 0,10 | 0,05 | 0,10 | 0,13 | - | 0,03 | 0,07 | 0,07 | 0,03 | 0,00 | 0,00 | 0,00 | 0,00 | 0,00 | 0,03 | 0,00 | 0,00 | 0,02 | 0,00 | 0,00 |
| **D6** | 0,03 | 0,12 | 0,00 | 0,00 | 0,03 | - | 0,06 | 0,03 | 0,18 | 0,02 | 0,11 | 0,01 | 0,01 | 0,00 | 0,07 | 0,07 | 0,03 | 0,00 | 0,01 | -0,09 |
| **D7** | 0,08 | 0,03 | 0,01 | 0,17 | 0,07 | 0,06 | - | 0,16 | 0,01 | 0,01 | 0,02 | 0,05 | 0,09 | 0,00 | 0,02 | 0,00 | 0,00 | 0,00 | 0,00 | -0,05 |
| **D8** | 0,00 | 0,03 | 0,05 | 0,02 | 0,07 | 0,03 | 0,16 | - | 0,14 | 0,06 | 0,04 | 0,01 | 0,00 | 0,00 | 0,05 | 0,00 | 0,00 | 0,02 | -0,01 | -0,02 |
| **D9** | 0,00 | 0,16 | 0,00 | 0,00 | 0,03 | 0,18 | 0,01 | 0,14 | - | 0,01 | 0,00 | 0,00 | 0,00 | 0,00 | 0,00 | 0,00 | 0,00 | 0,00 | 0,00 | -0,15 |
| **S1** | 0,00 | 0,01 | 0,00 | 0,00 | 0,00 | 0,02 | 0,01 | 0,06 | 0,01 | - | 0,21 | 0,12 | 0,00 | 0,25 | 0,09 | 0,00 | 0,00 | 0,11 | 0,00 | 0,00 |
| **S2** | 0,00 | 0,04 | 0,00 | 0,00 | 0,00 | 0,11 | 0,02 | 0,04 | 0,00 | 0,21 | - | 0,14 | 0,11 | 0,12 | 0,12 | 0,02 | 0,10 | 0,00 | 0,04 | -0,04 |
| **S3** | 0,00 | 0,01 | 0,01 | 0,05 | 0,00 | 0,01 | 0,05 | 0,01 | 0,00 | 0,12 | 0,14 | - | 0,11 | 0,04 | 0,13 | 0,00 | 0,00 | 0,00 | 0,00 | -0,04 |
| **S6** | 0,01 | 0,00 | 0,00 | 0,04 | 0,00 | 0,01 | 0,09 | 0,00 | 0,00 | 0,00 | 0,11 | 0,11 | - | 0,00 | 0,29 | 0,00 | 0,00 | 0,00 | 0,05 | -0,05 |
| **S9** | 0,00 | 0,00 | 0,00 | 0,00 | 0,00 | 0,00 | 0,00 | 0,00 | 0,00 | 0,25 | 0,12 | 0,04 | 0,00 | - | 0,01 | 0,00 | 0,00 | 0,06 | 0,00 | 0,00 |
| **S10** | 0,00 | 0,04 | 0,04 | 0,03 | 0,03 | 0,07 | 0,02 | 0,05 | 0,00 | 0,09 | 0,12 | 0,13 | 0,29 | 0,01 | - | 0,12 | 0,03 | 0,07 | 0,00 | -0,09 |
| **S4** | 0,00 | 0,04 | 0,00 | 0,00 | 0,00 | 0,07 | 0,00 | 0,00 | 0,00 | 0,00 | 0,02 | 0,00 | 0,00 | 0,00 | 0,12 | - | 0,23 | 0,10 | 0,11 | -0,10 |
| **S5** | 0,04 | 0,03 | 0,00 | 0,00 | 0,00 | 0,03 | 0,00 | 0,00 | 0,00 | 0,00 | 0,10 | 0,00 | 0,00 | 0,00 | 0,03 | 0,23 | - | 0,07 | 0,19 | -0,06 |
| **S7** | 0,02 | 0,00 | 0,00 | 0,00 | 0,02 | 0,00 | 0,00 | 0,02 | 0,00 | 0,11 | 0,00 | 0,00 | 0,00 | 0,06 | 0,07 | 0,10 | 0,07 | - | 0,03 | -0,02 |
| **S8** | 0,04 | 0,02 | 0,01 | 0,02 | 0,00 | 0,01 | 0,00 | -0,01 | 0,00 | 0,00 | 0,04 | 0,00 | 0,05 | 0,00 | 0,00 | 0,11 | 0,19 | 0,03 | - | -0,07 |
| **Q1** | -0,08 | -0,22 | -0,03 | -0,04 | 0,00 | -0,09 | -0,05 | -0,02 | -0,15 | 0,00 | -0,04 | -0,04 | -0,05 | 0,00 | -0,09 | -0,10 | -0,06 | -0,02 | -0,07 | - |

**Supplemental Table S2. Connection strengths between changes in individual depression and stress symptoms as well as changes in overall quality of life (underlying Figure 5).**

|  | **D1** | **D2** | **D3** | **D4** | **D5** | **D6** | **D7** | **D8** | **D9** | **S1** | **S2** | **S3** | **S6** | **S9** | **S10** | **S4** | **S5** | **S7** | **S8** | **Q1** |
| --- | --- | --- | --- | --- | --- | --- | --- | --- | --- | --- | --- | --- | --- | --- | --- | --- | --- | --- | --- | --- |
| **D1** | - | 0,25 | 0,01 | 0,08 | 0,00 | 0,02 | 0,02 | 0,00 | 0,00 | 0,04 | 0,03 | 0,02 | 0,00 | 0,09 | 0,01 | 0,00 | 0,05 | 0,00 | 0,00 | -0,09 |
| **D2** | 0,25 | - | 0,00 | 0,18 | 0,09 | 0,06 | 0,04 | 0,05 | 0,00 | 0,00 | 0,02 | 0,07 | 0,06 | 0,00 | 0,05 | 0,04 | 0,00 | 0,00 | 0,00 | -0,16 |
| **D3** | 0,01 | 0,00 | - | 0,22 | 0,15 | 0,00 | 0,01 | 0,06 | 0,00 | 0,00 | 0,00 | 0,09 | 0,01 | 0,00 | 0,00 | 0,00 | 0,03 | 0,00 | 0,00 | 0,00 |
| **D4** | 0,08 | 0,18 | 0,22 | - | 0,14 | 0,03 | 0,20 | 0,00 | -0,02 | 0,00 | 0,00 | 0,01 | 0,00 | 0,00 | 0,06 | 0,04 | 0,03 | 0,00 | 0,00 | -0,07 |
| **D5** | 0,00 | 0,09 | 0,15 | 0,14 | - | 0,05 | 0,03 | 0,03 | 0,03 | 0,00 | 0,00 | 0,00 | 0,09 | 0,00 | 0,00 | 0,00 | 0,00 | 0,01 | 0,00 | 0,00 |
| **D6** | 0,02 | 0,06 | 0,00 | 0,03 | 0,05 | - | 0,15 | 0,00 | 0,20 | 0,00 | 0,07 | 0,00 | 0,09 | -0,01 | 0,00 | 0,00 | 0,05 | 0,00 | 0,04 | -0,19 |
| **D7** | 0,02 | 0,04 | 0,01 | 0,20 | 0,03 | 0,15 | - | 0,09 | 0,00 | 0,03 | 0,01 | 0,11 | 0,05 | 0,01 | 0,00 | 0,03 | 0,00 | 0,00 | 0,03 | 0,00 |
| **D8** | 0,00 | 0,05 | 0,06 | 0,00 | 0,03 | 0,00 | 0,09 | - | 0,06 | 0,00 | 0,02 | 0,05 | 0,09 | 0,05 | 0,00 | 0,00 | 0,01 | 0,02 | 0,00 | 0,00 |
| **D9** | 0,00 | 0,00 | 0,00 | -0,02 | 0,03 | 0,20 | 0,00 | 0,06 | - | 0,00 | 0,00 | 0,00 | 0,00 | 0,00 | 0,07 | 0,05 | 0,02 | 0,05 | 0,00 | -0,02 |
| **S1** | 0,04 | 0,00 | 0,00 | 0,00 | 0,00 | 0,00 | 0,03 | 0,00 | 0,00 | - | 0,18 | 0,07 | 0,04 | 0,11 | 0,00 | 0,00 | 0,10 | 0,07 | 0,14 | 0,00 |
| **S2** | 0,03 | 0,02 | 0,00 | 0,00 | 0,00 | 0,07 | 0,01 | 0,02 | 0,00 | 0,18 | - | 0,16 | 0,12 | 0,11 | 0,18 | 0,00 | 0,11 | 0,00 | 0,00 | -0,03 |
| **S3** | 0,02 | 0,07 | 0,09 | 0,01 | 0,00 | 0,00 | 0,11 | 0,05 | 0,00 | 0,07 | 0,16 | - | 0,06 | 0,01 | 0,14 | 0,04 | 0,00 | 0,00 | 0,00 | -0,12 |
| **S6** | 0,00 | 0,06 | 0,01 | 0,00 | 0,09 | 0,09 | 0,05 | 0,09 | 0,00 | 0,04 | 0,12 | 0,06 | - | 0,01 | 0,20 | 0,00 | 0,03 | 0,00 | 0,00 | -0,01 |
| **S9** | 0,09 | 0,00 | 0,00 | 0,00 | 0,00 | -0,01 | 0,01 | 0,05 | 0,00 | 0,11 | 0,11 | 0,01 | 0,01 | - | 0,05 | 0,00 | 0,00 | 0,07 | 0,03 | -0,03 |
| **S10** | 0,01 | 0,05 | 0,00 | 0,06 | 0,00 | 0,00 | 0,00 | 0,00 | 0,07 | 0,00 | 0,18 | 0,14 | 0,20 | 0,05 | - | 0,11 | 0,00 | 0,15 | 0,02 | -0,09 |
| **S4** | 0,00 | 0,04 | 0,00 | 0,04 | 0,00 | 0,00 | 0,03 | 0,00 | 0,05 | 0,00 | 0,00 | 0,04 | 0,00 | 0,00 | 0,11 | - | 0,25 | 0,06 | 0,07 | -0,13 |
| **S5** | 0,05 | 0,00 | 0,03 | 0,03 | 0,00 | 0,05 | 0,00 | 0,01 | 0,02 | 0,10 | 0,11 | 0,00 | 0,03 | 0,00 | 0,00 | 0,25 | - | 0,04 | 0,16 | -0,02 |
| **S7** | 0,00 | 0,00 | 0,00 | 0,00 | 0,01 | 0,00 | 0,00 | 0,02 | 0,05 | 0,07 | 0,00 | 0,00 | 0,00 | 0,07 | 0,15 | 0,06 | 0,04 | - | 0,12 | 0,00 |
| **S8** | 0,00 | 0,00 | 0,00 | 0,00 | 0,00 | 0,04 | 0,03 | 0,00 | 0,00 | 0,14 | 0,00 | 0,00 | 0,00 | 0,03 | 0,02 | 0,07 | 0,16 | 0,12 | - | -0,07 |
| **Q1** | -0,09 | -0,16 | 0,00 | -0,07 | 0,00 | -0,19 | 0,00 | 0,00 | -0,02 | 0,00 | -0,03 | -0,12 | -0,01 | -0,03 | -0,09 | -0,13 | -0,02 | 0,00 | -0,07 | - |
